# 3-(2,4-Difluoro­anilino)-9-nitro­dibenzo[*b*,*e*]oxepin-11(6*H*)-one

**DOI:** 10.1107/S1600536811002881

**Published:** 2011-02-02

**Authors:** Benjamin Baur, Dieter Schollmeyer, Stefan Laufer

**Affiliations:** a Institute of Pharmacy, Department of Pharmaceutical Chemistry, Eberhard Karls University Tübingen, Auf der Morgenstelle 8, 72076 Tübingen, Germany; b Department of Organic Chemistry, Johannes Gutenberg-University Mainz, Duessbergweg 10-14, 55099 Mainz, Germany

## Abstract

In the title compound, C_20_H_12_F_2_N_2_O_4_, the two benzene rings of the tricyclic unit are oriented at a dihedral angle of 30.6 (1)°. The 2,4-difluoro­anilino residue is oriented at a dihedral angle of 68.2 (1)° with respect to the phen­oxy ring. In the crystal, N—H⋯O hydrogen bonds between the amino group and the carbonyl O atom of the oxepinone ring link the mol­ecules into infinte chains along the *c* axis.

## Related literature

For palladium-catalysed amination reactions of aryl halides with anilines, see: Jensen *et al.* (2004 ▶). For p38 MAP kinase inhibitors based on dibenzo[*b*,*e*]oxepin-11(6*H*)-one, see: Laufer *et al.* (2006 ▶).
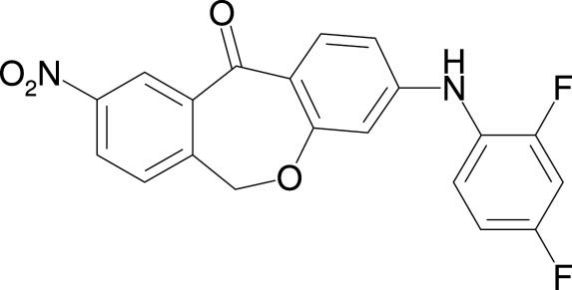


## Experimental

### 

#### Crystal data

C_20_H_12_F_2_N_2_O_4_
*M* *_r_* = 382.32
Orthorhombic, 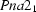

*a* = 27.0813 (15) Å
*b* = 13.0411 (8) Å
*c* = 4.5998 (2) Å
*V* = 1624.51 (15) Å^3^
*Z* = 4
Cu *K*α radiation
μ = 1.08 mm^−1^
*T* = 193 K
0.47 × 0.24 × 0.12 mm

#### Data collection

Enraf–Nonius CAD-4 diffractometer
Absorption correction: numerical (*CORINC*; Dräger & Gattow, 1971 ▶) *T*_min_ = 0.721, *T*_max_ = 0.882
3417 measured reflections
3010 independent reflections
2924 reflections with *I* > 2σ(*I*)
*R*_int_ = 0.051
3 standard reflections every 60 min  intensity decay: 3%

#### Refinement

*R*[*F* ^2^ > 2σ(*F* ^2^)] = 0.047
*wR*(*F* ^2^) = 0.127
*S* = 1.03
3010 reflections
253 parameters
1 restraint
H-atom parameters constrained
Δρ_max_ = 0.17 e Å^−3^
Δρ_min_ = −0.20 e Å^−3^
Absolute structure: Flack, (1983 ▶), 1270 Friedel pairs
Flack parameter: −0.22 (18)

### 

Data collection: *CAD-4 Software* (Enraf–Nonius, 1989 ▶); cell refinement: *CAD-4 Software*; data reduction: *CORINC* (Dräger & Gattow, 1971 ▶); program(s) used to solve structure: *SIR97* (Altomare *et al.*, 1999 ▶); program(s) used to refine structure: *SHELXL97* (Sheldrick, 2008 ▶); molecular graphics: *PLATON* (Spek, 2009 ▶); software used to prepare material for publication: *PLATON*.

## Supplementary Material

Crystal structure: contains datablocks I, global. DOI: 10.1107/S1600536811002881/im2261sup1.cif

Structure factors: contains datablocks I. DOI: 10.1107/S1600536811002881/im2261Isup2.hkl

Additional supplementary materials:  crystallographic information; 3D view; checkCIF report

## Figures and Tables

**Table 1:** Hydrogen-bond geometry (Å, °)

*D*—H⋯*A*	*D*—H	H⋯*A*	*D*⋯*A*	*D*—H⋯*A*
N16—H16⋯O25^i^	0.95	2.32	3.236 (3)	162

Symmetry code: (i) 
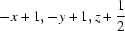
.

**Fig. 1.**
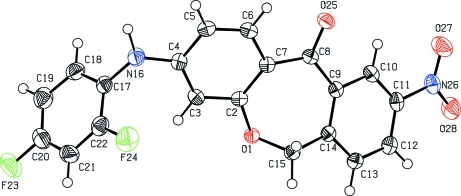
View of compound I. Displacement ellipsoids are drawn at the 50% probability level.

## supplementary crystallographic information

### Comment

Based on dibenzo[b,e]oxepin-11(6*H*)-one (Laufer *et al.* 2006) as p38 MAP kinase inhibitors, our intent was to synthesize disubstituted oxepin derivatives. The title compound was synthesized in the course of an ongoing study to increase the solubility of the molecules. The structure of the title compound, at 193 K shows orthorhombic symmetry. The two phenyl rings of the tricyclic unit are oriented at a dihedral angle of 30.6 (1°). The 2,4-difluoroanilino residue is oriented at a dihedral angel of 68.2 (1°) towards the phenoxy ring. The crystal stucture is characterized by an intermolecular hydrogen bond N16–H···O25 (2.32 Å).

### Experimental

For the preperation of the title compound a mixture of 200 mg (0.69 mmol) 3-chloro-9-nitrodibenzo[b,e]oxepin-11(6*H*)-one, 100 mg (0.77 mmol) 2–4-difluoroaniline, 1.00 g (3.07 mmol) Cs_2_CO_3_, 45 mg (0.10 mmol) 2-(dicyclohexylphosphino)-2`-4`-6`-triisopropylbiphenyl and 20 mg (0.09 mmol) Pd(OAc)_2_ in 2 ml absolute *tert*-butanol and 10 ml absolute 2.4-dioxane was stirred for 1 h at 284 K under an argon atmosphere. The mixture was then filtered and evaporated under reduced pressure. The residue was purified by flash chromatography (SiO_2_ n-hexane/ethyl acetate 2:1) to get 103 mg (0,27 mmol) of the product as a yellow solid (yield 39 %). Crystals of the title compound were obtained by slow evaporation of diethyl ether and hexane (1:1) at room temperature.

### Refinement

Hydrogen atoms attached to carbons were placed at calculated positions with C—H = 0.95 Å (aromatic) or 0.98–0.99 Å (*sp*^3^ C-atom). Hydrogen atom attached to nitrogen was located in diff. Fourier maps. All H atoms were refined in the riding-model approximation with isotropic displacement parameters (set at 1.2–1.5 times of the *U*_eq_ of the parent atom). The absolute structure was determined on the basis of 1270 Friedel pairs.

### Crystal data

**Table:**

C_20_H_12_F_2_N_2_O_4_	*F*(000) = 784
*M**_r_* = 382.32	*D*_x_ = 1.563 Mg m^−^^3^
Orthorhombic, *P**n**a*2_1_	Cu *K*α radiation, λ = 1.54178 Å
Hall symbol: P 2c -2n	Cell parameters from 25 reflections
*a* = 27.0813 (15) Å	θ = 65–69°
*b* = 13.0411 (8) Å	µ = 1.08 mm^−^^1^
*c* = 4.5998 (2) Å	*T* = 193 K
*V* = 1624.51 (15) Å^3^	Needle, yellow
*Z* = 4	0.47 × 0.24 × 0.12 mm

### Data collection

**Table:**

Enraf–Nonius CAD-4 diffractometer	2924 reflections with *I* > 2σ(*I*)
Radiation source: rotating anode	*R*_int_ = 0.051
graphite	θ_max_ = 69.8°, θ_min_ = 3.3°
ω/2θ scans	*h* = −33→33
Absorption correction: numerical (CORINC; Dräger & Gattow, 1971)	*k* = −15→15
*T*_min_ = 0.721, *T*_max_ = 0.882	*l* = −5→5
3417 measured reflections	3 standard reflections every 60 min
3010 independent reflections	intensity decay: 3%

### Refinement

**Table:**

Refinement on *F*^2^	Secondary atom site location: difference Fourier map
Least-squares matrix: full	Hydrogen site location: inferred from neighbouring sites
*R*[*F*^2^ > 2σ(*F*^2^)] = 0.047	H-atom parameters constrained
*wR*(*F*^2^) = 0.127	*w* = 1/[σ^2^(*F*_o_^2^) + (0.0989*P*)^2^ + 0.3414*P*] where *P* = (*F*_o_^2^ + 2*F*_c_^2^)/3
*S* = 1.03	(Δ/σ)_max_ = 0.023
3010 reflections	Δρ_max_ = 0.17 e Å^−^^3^
253 parameters	Δρ_min_ = −0.20 e Å^−^^3^
1 restraint	Absolute structure: Flack, (1983), 1270 Friedel pairs
Primary atom site location: structure-invariant direct methods	Flack parameter: −0.22 (18)

### Special details

**Table:**

Experimental. ^1^H NMR (200 MHz, DMSO-d~6~) δ in p.p.m. 5.32 (s, 2 H), 6.26 (m, 1 H), 6.65 (dd, *J*=8.65, 1.96 Hz, 1 H), 7.11 (m, 1 H), 7.39 (m, 2 H), 7.82 (m, 1 H), 8.02 (d, *J*=8.97 Hz, 1 H), 8.41 (dd, *J*=8.21, 2.53 Hz, 1 H), 8.52 (d, *J*=2.40 Hz, 1 H), 8.85 (s, NH,1 H).^13^C NMR (50 MHz, DMSO-d~6~) δ in p.p.m. 72.5, 102.1, 110.5, 116.5, 124.3, 127.0, 130.4, 134.1, 141.1, 142.9, 148.3, 152.9, 163.4, 185.1, C—F not detected.GC/MS, *m/z* (%) 382 (100, *M*^+^), 308 (12), 152 (9), 98 (7), 63 (1).
Geometry. All e.s.d.'s (except the e.s.d. in the dihedral angle between two l.s. planes) are estimated using the full covariance matrix. The cell e.s.d.'s are taken into account individually in the estimation of e.s.d.'s in distances, angles and torsion angles; correlations between e.s.d.'s in cell parameters are only used when they are defined by crystal symmetry. An approximate (isotropic) treatment of cell e.s.d.'s is used for estimating e.s.d.'s involving l.s. planes.
Refinement. Refinement of *F*^2^ against ALL reflections. The weighted *R*-factor *wR* and goodness of fit *S* are based on *F*^2^, conventional *R*-factors *R* are based on *F*, with *F* set to zero for negative *F*^2^. The threshold expression of *F*^2^ > σ(*F*^2^) is used only for calculating *R*-factors(gt) *etc*. and is not relevant to the choice of reflections for refinement. *R*-factors based on *F*^2^ are statistically about twice as large as those based on *F*, and *R*- factors based on ALL data will be even larger.

### Fractional atomic coordinates and isotropic or equivalent isotropic displacement parameters (Å^2^)

**Table:**

	*x*	*y*	*z*	*U*_iso_*/*U*_eq_	
O1	0.58864 (5)	0.24963 (11)	0.3890 (3)	0.0240 (3)	
C2	0.56177 (7)	0.30020 (15)	0.5965 (4)	0.0215 (4)	
C3	0.51656 (8)	0.25513 (16)	0.6587 (5)	0.0242 (4)	
H3	0.5068	0.1947	0.5587	0.029*	
C4	0.48548 (8)	0.29789 (17)	0.8665 (5)	0.0245 (4)	
C5	0.50161 (8)	0.38484 (16)	1.0212 (5)	0.0242 (4)	
H5	0.4818	0.4123	1.1728	0.029*	
C6	0.54556 (8)	0.42947 (15)	0.9536 (5)	0.0234 (4)	
H6	0.5556	0.4882	1.0605	0.028*	
C7	0.57698 (7)	0.39218 (15)	0.7306 (4)	0.0216 (4)	
C8	0.61655 (7)	0.46277 (15)	0.6377 (4)	0.0222 (4)	
C9	0.65513 (7)	0.43587 (15)	0.4150 (5)	0.0215 (4)	
C10	0.68194 (8)	0.51865 (17)	0.3032 (5)	0.0248 (4)	
H10	0.6752	0.5865	0.3670	0.030*	
C11	0.71823 (7)	0.50081 (16)	0.0997 (5)	0.0262 (5)	
C12	0.73012 (8)	0.40368 (18)	0.0000 (5)	0.0275 (5)	
H12	0.7551	0.3936	−0.1423	0.033*	
C13	0.70420 (7)	0.32175 (17)	0.1161 (5)	0.0253 (5)	
H13	0.7120	0.2541	0.0550	0.030*	
C14	0.66696 (7)	0.33663 (16)	0.3204 (5)	0.0223 (4)	
C15	0.64067 (7)	0.24498 (15)	0.4420 (5)	0.0253 (5)	
H15A	0.6542	0.1820	0.3525	0.030*	
H15B	0.6466	0.2412	0.6541	0.030*	
N16	0.43947 (7)	0.25975 (16)	0.9317 (5)	0.0341 (5)	
H16	0.4191	0.3040	1.0435	0.041*	
C17	0.41597 (8)	0.18098 (18)	0.7713 (5)	0.0287 (5)	
C18	0.37502 (8)	0.20134 (19)	0.5998 (6)	0.0352 (5)	
H18	0.3631	0.2697	0.5860	0.042*	
C19	0.35112 (10)	0.1240 (2)	0.4481 (7)	0.0434 (6)	
H19	0.3226	0.1381	0.3345	0.052*	
C20	0.36991 (10)	0.0266 (2)	0.4670 (6)	0.0407 (6)	
C21	0.41046 (10)	0.00166 (19)	0.6308 (7)	0.0409 (6)	
H21	0.4229	−0.0664	0.6384	0.049*	
C22	0.43238 (9)	0.0805 (2)	0.7842 (6)	0.0355 (6)	
F23	0.34769 (7)	−0.04940 (15)	0.3148 (5)	0.0629 (6)	
F24	0.47133 (6)	0.05902 (13)	0.9539 (5)	0.0558 (5)	
O25	0.61751 (6)	0.55042 (11)	0.7364 (4)	0.0302 (4)	
N26	0.74461 (7)	0.58919 (15)	−0.0221 (5)	0.0315 (4)	
O27	0.73855 (7)	0.67347 (14)	0.0898 (5)	0.0476 (5)	
O28	0.77170 (7)	0.57374 (15)	−0.2321 (5)	0.0434 (5)	

### Atomic displacement parameters (Å^2^)

**Table:**

	*U*^11^	*U*^22^	*U*^33^	*U*^12^	*U*^13^	*U*^23^
O1	0.0251 (7)	0.0263 (7)	0.0205 (8)	−0.0019 (5)	0.0021 (6)	−0.0047 (6)
C2	0.0275 (9)	0.0227 (10)	0.0143 (9)	0.0030 (7)	−0.0011 (8)	0.0020 (8)
C3	0.0306 (10)	0.0249 (10)	0.0171 (10)	−0.0024 (8)	−0.0015 (9)	−0.0011 (8)
C4	0.0270 (10)	0.0287 (10)	0.0179 (11)	−0.0006 (8)	0.0009 (8)	0.0029 (8)
C5	0.0303 (10)	0.0286 (11)	0.0137 (10)	0.0044 (8)	0.0011 (8)	0.0012 (8)
C6	0.0327 (10)	0.0243 (9)	0.0132 (10)	0.0013 (8)	−0.0043 (8)	0.0005 (8)
C7	0.0253 (9)	0.0251 (10)	0.0142 (10)	0.0013 (8)	−0.0036 (8)	0.0021 (8)
C8	0.0271 (9)	0.0210 (9)	0.0184 (10)	0.0019 (7)	−0.0060 (8)	0.0015 (8)
C9	0.0230 (9)	0.0245 (9)	0.0170 (10)	−0.0001 (8)	−0.0063 (8)	0.0033 (8)
C10	0.0283 (10)	0.0227 (9)	0.0234 (11)	−0.0015 (8)	−0.0083 (9)	0.0037 (8)
C11	0.0246 (9)	0.0300 (11)	0.0239 (11)	−0.0059 (8)	−0.0052 (8)	0.0067 (9)
C12	0.0252 (9)	0.0333 (11)	0.0239 (11)	−0.0003 (8)	−0.0023 (9)	0.0038 (9)
C13	0.0256 (9)	0.0261 (10)	0.0241 (11)	0.0017 (8)	−0.0032 (9)	0.0014 (8)
C14	0.0218 (9)	0.0249 (10)	0.0201 (10)	0.0013 (7)	−0.0055 (8)	0.0019 (8)
C15	0.0266 (10)	0.0220 (10)	0.0275 (11)	0.0005 (7)	0.0022 (9)	0.0028 (9)
N16	0.0318 (9)	0.0404 (11)	0.0301 (11)	−0.0050 (8)	0.0088 (9)	−0.0101 (9)
C17	0.0274 (10)	0.0336 (11)	0.0250 (12)	−0.0039 (8)	0.0078 (9)	−0.0015 (9)
C18	0.0338 (11)	0.0355 (13)	0.0363 (14)	0.0036 (9)	0.0046 (10)	−0.0016 (11)
C19	0.0371 (12)	0.0532 (15)	0.0400 (16)	−0.0028 (11)	−0.0025 (12)	−0.0075 (13)
C20	0.0447 (14)	0.0424 (13)	0.0349 (15)	−0.0147 (11)	0.0132 (11)	−0.0110 (11)
C21	0.0444 (12)	0.0287 (12)	0.0495 (16)	−0.0035 (10)	0.0142 (12)	0.0015 (12)
C22	0.0309 (11)	0.0381 (12)	0.0373 (14)	−0.0009 (9)	0.0052 (11)	0.0077 (11)
F23	0.0677 (11)	0.0604 (11)	0.0606 (12)	−0.0257 (9)	0.0120 (10)	−0.0305 (10)
F24	0.0483 (9)	0.0541 (10)	0.0649 (12)	0.0051 (7)	−0.0134 (9)	0.0171 (9)
O25	0.0352 (8)	0.0244 (8)	0.0311 (9)	−0.0034 (6)	0.0017 (7)	−0.0047 (7)
N26	0.0312 (9)	0.0318 (10)	0.0314 (11)	−0.0070 (8)	−0.0010 (9)	0.0060 (9)
O27	0.0584 (11)	0.0300 (9)	0.0545 (13)	−0.0103 (8)	0.0147 (10)	0.0022 (8)
O28	0.0460 (9)	0.0441 (10)	0.0402 (11)	−0.0120 (8)	0.0137 (9)	0.0031 (9)

### Geometric parameters (Å, °)

**Table:**

O1—C2	1.370 (2)	C12—H12	0.9500
O1—C15	1.431 (3)	C13—C14	1.392 (3)
C2—C3	1.388 (3)	C13—H13	0.9500
C2—C7	1.410 (3)	C14—C15	1.499 (3)
C3—C4	1.390 (3)	C15—H15A	0.9900
C3—H3	0.9500	C15—H15B	0.9900
C4—N16	1.375 (3)	N16—C17	1.416 (3)
C4—C5	1.408 (3)	N16—H16	0.9504
C5—C6	1.361 (3)	C17—C18	1.387 (3)
C5—H5	0.9500	C17—C22	1.385 (3)
C6—C7	1.419 (3)	C18—C19	1.387 (4)
C6—H6	0.9500	C18—H18	0.9500
C7—C8	1.476 (3)	C19—C20	1.371 (4)
C8—O25	1.230 (3)	C19—H19	0.9500
C8—C9	1.504 (3)	C20—F23	1.355 (3)
C9—C10	1.399 (3)	C20—C21	1.371 (4)
C9—C14	1.403 (3)	C21—C22	1.381 (4)
C10—C11	1.377 (3)	C21—H21	0.9500
C10—H10	0.9500	C22—F24	1.341 (3)
C11—C12	1.385 (3)	N26—O27	1.225 (3)
C11—N26	1.467 (3)	N26—O28	1.230 (3)
C12—C13	1.385 (3)		
			
C2—O1—C15	115.13 (16)	C12—C13—H13	119.3
O1—C2—C3	114.11 (19)	C14—C13—H13	119.3
O1—C2—C7	123.99 (18)	C13—C14—C9	120.24 (19)
C3—C2—C7	121.85 (19)	C13—C14—C15	119.00 (19)
C2—C3—C4	120.4 (2)	C9—C14—C15	120.75 (19)
C2—C3—H3	119.8	O1—C15—C14	111.72 (16)
C4—C3—H3	119.8	O1—C15—H15A	109.3
N16—C4—C3	123.6 (2)	C14—C15—H15A	109.3
N16—C4—C5	117.53 (19)	O1—C15—H15B	109.3
C3—C4—C5	118.85 (19)	C14—C15—H15B	109.3
C6—C5—C4	120.02 (19)	H15A—C15—H15B	107.9
C6—C5—H5	120.0	C4—N16—C17	123.8 (2)
C4—C5—H5	120.0	C4—N16—H16	115.2
C5—C6—C7	122.89 (19)	C17—N16—H16	117.5
C5—C6—H6	118.6	C18—C17—C22	117.5 (2)
C7—C6—H6	118.6	C18—C17—N16	121.1 (2)
C2—C7—C6	115.63 (18)	C22—C17—N16	121.3 (2)
C2—C7—C8	128.01 (18)	C17—C18—C19	121.4 (2)
C6—C7—C8	115.54 (18)	C17—C18—H18	119.3
O25—C8—C7	119.20 (19)	C19—C18—H18	119.3
O25—C8—C9	116.95 (18)	C20—C19—C18	117.9 (2)
C7—C8—C9	123.78 (18)	C20—C19—H19	121.0
C10—C9—C14	118.6 (2)	C18—C19—H19	121.0
C10—C9—C8	115.52 (18)	F23—C20—C19	118.7 (3)
C14—C9—C8	125.81 (18)	F23—C20—C21	117.8 (3)
C11—C10—C9	119.3 (2)	C19—C20—C21	123.5 (2)
C11—C10—H10	120.3	C20—C21—C22	116.6 (2)
C9—C10—H10	120.3	C20—C21—H21	121.7
C10—C11—C12	123.1 (2)	C22—C21—H21	121.7
C10—C11—N26	118.3 (2)	F24—C22—C21	118.7 (2)
C12—C11—N26	118.6 (2)	F24—C22—C17	118.3 (2)
C11—C12—C13	117.4 (2)	C21—C22—C17	123.0 (2)
C11—C12—H12	121.3	O27—N26—O28	123.8 (2)
C13—C12—H12	121.3	O27—N26—C11	118.7 (2)
C12—C13—C14	121.3 (2)	O28—N26—C11	117.5 (2)
			
C15—O1—C2—C3	−140.97 (18)	C12—C13—C14—C9	−0.5 (3)
C15—O1—C2—C7	41.3 (3)	C12—C13—C14—C15	−179.1 (2)
O1—C2—C3—C4	179.62 (18)	C10—C9—C14—C13	−0.9 (3)
C7—C2—C3—C4	−2.6 (3)	C8—C9—C14—C13	−178.83 (19)
C2—C3—C4—N16	177.5 (2)	C10—C9—C14—C15	177.64 (19)
C2—C3—C4—C5	−2.8 (3)	C8—C9—C14—C15	−0.3 (3)
N16—C4—C5—C6	−176.1 (2)	C2—O1—C15—C14	−88.1 (2)
C3—C4—C5—C6	4.2 (3)	C13—C14—C15—O1	−121.5 (2)
C4—C5—C6—C7	−0.2 (3)	C9—C14—C15—O1	59.9 (3)
O1—C2—C7—C6	−176.19 (18)	C3—C4—N16—C17	−8.2 (4)
C3—C2—C7—C6	6.3 (3)	C5—C4—N16—C17	172.1 (2)
O1—C2—C7—C8	14.7 (3)	C4—N16—C17—C18	−110.1 (3)
C3—C2—C7—C8	−162.8 (2)	C4—N16—C17—C22	71.2 (3)
C5—C6—C7—C2	−4.9 (3)	C22—C17—C18—C19	0.4 (4)
C5—C6—C7—C8	165.57 (19)	N16—C17—C18—C19	−178.4 (2)
C2—C7—C8—O25	162.9 (2)	C17—C18—C19—C20	−1.5 (4)
C6—C7—C8—O25	−6.2 (3)	C18—C19—C20—F23	−178.5 (2)
C2—C7—C8—C9	−14.0 (3)	C18—C19—C20—C21	1.0 (4)
C6—C7—C8—C9	176.95 (17)	F23—C20—C21—C22	−179.9 (2)
O25—C8—C9—C10	−11.8 (3)	C19—C20—C21—C22	0.6 (4)
C7—C8—C9—C10	165.16 (18)	C20—C21—C22—F24	178.1 (2)
O25—C8—C9—C14	166.2 (2)	C20—C21—C22—C17	−1.8 (4)
C7—C8—C9—C14	−16.9 (3)	C18—C17—C22—F24	−178.6 (2)
C14—C9—C10—C11	1.4 (3)	N16—C17—C22—F24	0.1 (3)
C8—C9—C10—C11	179.54 (17)	C18—C17—C22—C21	1.3 (4)
C9—C10—C11—C12	−0.6 (3)	N16—C17—C22—C21	−179.9 (2)
C9—C10—C11—N26	177.82 (18)	C10—C11—N26—O27	10.3 (3)
C10—C11—C12—C13	−0.8 (3)	C12—C11—N26—O27	−171.3 (2)
N26—C11—C12—C13	−179.2 (2)	C10—C11—N26—O28	−169.8 (2)
C11—C12—C13—C14	1.3 (3)	C12—C11—N26—O28	8.7 (3)

### Hydrogen-bond geometry (Å, °)

**Table:**

*D*—H···*A*	*D*—H	H···*A*	*D*···*A*	*D*—H···*A*
N16—H16···O25^i^	0.95	2.32	3.236 (3)	162

Symmetry codes: (i) −*x*+1, −*y*+1, *z*+1/2.
